# The relationship between SIRT1 and inflammation: a systematic review and meta-analysis

**DOI:** 10.3389/fimmu.2024.1465849

**Published:** 2024-11-29

**Authors:** Haiyang Sun, Dong Li, Chaojie Wei, Liping Liu, Zhuoyuan Xin, Hang Gao, Rong Gao

**Affiliations:** ^1^ Department of Immunology, College of Basic Medical Sciences, Jilin University, Changchun, China; ^2^ Key Laboratory of Zoonosis Research, Ministry of Education, College of Basic Medical Science, Jilin University, Changchun, China; ^3^ Department of Bone and Joint Surgery, Orthopaedic Surgery Center, The First Hospital of Jilin University, Changchun, China; ^4^ Department of Respiratory and Critical Care Medicine, The Second Hospital of Jilin University, Changchun, China

**Keywords:** SIRT1, inflammation, meta-analysis, NF-κB, systematic review

## Abstract

Recent studies underscore the anti-inflammatory role of SIRT1; however, its levels during inflammatory states remain ambiguous. We synthesized relevant studies up to 20 March 2024 to evaluate the relationship between SIRT1 and inflammation, using data from three major databases. Employing a random-effects model, we analyzed both cross-sectional and longitudinal studies, calculating weighted mean differences (WMDs) for pooled effect sizes. Subgroup and sensitivity analyses, along with a risk of bias assessment, were also conducted. We reviewed 13 publications, encompassing 21 datasets and 2,028 participants. The meta-analysis indicated higher SIRT1 levels in inflammatory groups compared to control groups pre-adjustment (WMD, 3.18 ng/ml; 95% CI 2.30, 4.06 ng/ml; *P*<0.001; *I²*= 99.7%) and post-adjustment (WMD, 0.88 ng/ml; 95% CI 0.14, 1.62 ng/ml; *P*<0.001; *I²*= 99.5%). Notably, middle-aged patients with inflammation exhibited lower SIRT1 levels (WMD, −0.85 ng/ml; 95% CI −1.47, −0.22 ng/ml; *P*= 0.008; *I²*= 95.4%), while groups characterized by East Asian descent, plasma studies, autoimmune conditions, and musculoskeletal disorders showed higher levels. The findings suggest that inflammation generally upregulates SIRT1, potentially elucidating its role in immunobiological processes. However, the significant heterogeneity observed, partly due to the cross-sectional nature of some data, limits insights into the duration of disease progression, which remains highly variable.

## 1 Introduction

SIRT1 (Silent Information Regulator 2 Homolog 1) is a protein that is part of the Sirtuin family, which comprises nicotinamide adenine dinucleotide (NAD) dependent deacetylases. This protein is encoded by the SIRT1 gene and is instrumental in maintaining cellular homeostasis (1). It plays pivotal roles in a variety of biological processes such as cellular metabolism (2–5), aging (6), DNA repair (7), inflammation (8), and oxidative stress (8–10). One key function of SIRT1 is its ability to regulate cellular metabolism. As a deacetylase, SIRT1 removes acetyl groups from various target proteins, including transcription factors and coactivators. This action allows SIRT1 to influence critical cellular energy metabolism pathways (11), including those involved in glucose and fatty acid metabolism (5, 12) as well as insulin signaling (13). SIRT1 also plays a significant role in the regulation of aging (14) and longevity. It has been shown to affect lifespan in various organisms including yeast, worms, flies, and mice. SIRT1 influences several key signaling pathways associated with aging, such as the FOXO (15), p53 (16), and AMPK (17) pathways. Through its regulatory effects on these pathways, SIRT1 helps to control cellular senescence and promotes longevity.

Moreover, SIRT1 is involved in modulating inflammation and stress responses. It can regulate the activity of transcription factors that are key to the inflammatory response, such as NF-κB (18), and can alter the production of pro-inflammatory cytokines. Additionally, SIRT1 plays a role in pathways related to cellular stress, including those involved in oxidative stress and DNA damage repair. The dysregulation of SIRT1 has been linked to a variety of diseases, including metabolic disorders (19), neurodegenerative diseases (20), cardiovascular diseases (21), and cancer (22), highlighting its critical function in human health and disease.

Some studies have indicated a potential connection between inflammation and SIRT1 levels (23–32, 58, 69, 70). However, there has been no comprehensive analysis of the relationship between SIRT1 and inflammation. Therefore, this article aims to outline the known immunobiological functions of SIRT1 in the onset and progression of inflammation, and to conduct a meta-analysis of published studies concerning the link between SIRT1 and inflammatory diseases.

## 2 Systematic review

### 2.1 SIRT1 biological function

SIRT1, originally characterized as an NAD+-dependent histone deacetylase (33), catalyzes the removal of acetyl groups from specific lysine residues on various histones, such as H1K26, H4K16, H3K9, and H3K14. This deacetylation process is crucial for modulating gene expression by promoting chromatin condensation and silencing specific genes, thereby influencing a multitude of biological processes (34). Beyond its role in histone modification, SIRT1 exhibits a broader spectrum of biological functions through the deacetylation of non-histone proteins. These proteins include a diverse range of cellular components like transcription factors, nuclear receptors, enzymes crucial for metabolic pathways, cell signaling molecules, and elements involved in DNA repair. By targeting these varied proteins, SIRT1 acts as a key regulator of cellular energy status and provides protection against multiple stressors, including metabolic imbalances, oxidative stress, genotoxic damage, and oncogenic pressures. Consequently, SIRT1 plays a vital role in maintaining cellular homeostasis and preventing the progression of various diseases.

SIRT1, a crucial metabolic regulator and a key target for pathways such as the mammalian target of rapamycin (mTOR) and AMPK, plays a vital role in maintaining metabolic homeostasis (35). As an essential energy sensor, SIRT1 orchestrates metabolic pathways and facilitates physiological responses to dietary changes. During calorie restriction or fasting, when blood glucose levels decline, hepatic metabolism shifts toward glycogen breakdown and gluconeogenesis to maintain a consistent glucose supply. In this state, liver cells upregulate SIRT1 expression and activity, which enhances the deacetylation of transcriptional co-activators such as PGC-1α and FOXO1 (36), thereby activating them. This activation promotes the transcription of genes involved in gluconeogenesis while simultaneously suppressing those related to glycolysis. Additionally, SIRT1 influences lipid metabolism by deacetylating the liver X receptor, leading to the downregulation of protein-tyrosine phosphatase 1B (PTP1B) (37). This alteration facilitates increased reverse cholesterol transport and reduces insulin resistance.

In pancreatic β-cells, SIRT1 enhances glucose-stimulated insulin production and secretion. In white adipose tissue, it promotes fat mobilization by suppressing PPARγ through interaction with corepressor proteins NCOR and SMRT (38). Heightened SIRT1 levels during caloric restriction contribute to enhanced insulin sensitivity, reduced cholesterol absorption, and decreased fat storage. In muscle tissue, SIRT1 activates PGC-1α during fasting, which subsequently drives PPARα-mediated transcription of genes associated with mitochondrial oxidative phosphorylation and electron transport chain processes (39). This activation enables efficient synchronization of fatty acid oxidation with energy production, thereby preserving glucose within the cell for other vital functions.

Furthermore, SIRT1 plays a crucial role in regulating mitochondrial function. It acts as a functional regulator of the NF-κB-dependent signal transducer and activator of transcription 3 (STAT3), a cytokine-responsive transcription factor that promotes mitochondrial biogenesis (40). STAT3 enhances oxidative phosphorylation by interacting directly with complex I of the electron transport chain within mitochondria. Research has shown that the absence of SIRT1 in murine embryonic fibroblast (MEF) cells results in increased mitochondrial respiration compared to their wild-type counterparts. This increased activity is evidenced by elevated oxygen consumption rates, heightened enzyme activities of electron transport chain complexes, and higher production of ATP and lactate.

Mitochondrial biogenesis is a complex process requiring the coordinated expression of both nuclear-encoded and mitochondrial-encoded genes. SIRT1, through the activation of PGC-1α, orchestrates this process by regulating the expression of mitochondrial genes in both the nucleus and mitochondria (41). However, evidence also suggests that SIRT1 may have a dual effect on PGC-1α transcription. In certain contexts, it might suppress PGC-1α transcription by deacetylating STAT3, thus balancing mitochondrial biogenesis with other cellular processes. The intricate interplay between SIRT1, STAT3, and PGC-1α underscores the multifaceted regulatory network modulating mitochondrial function and overall energy metabolism.

SIRT1 plays a significant role in various apoptosis pathways, including those involving P53, FOXO, and NF-κB transcription factors. SIRT1 influences these pathways by deacetylating these proteins, thereby modulating their activity. In the case of P53, SIRT1 deacetylates this tumor suppressor protein, reducing its DNA-binding ability and consequently protecting cells from P53-mediated apoptosis. In instances where SIRT1 is deficient, P53 becomes more acetylated, increasing the likelihood of apoptosis following exposure to ionizing radiation (IR). SIRT1 also targets the FOXO family of Forkhead transcription factors, which are crucial for sensing insulin signaling and regulating longevity. By deacetylating FOXO proteins, SIRT1 enhances FOXO3’s ability to induce cell cycle arrest and resistance to oxidative stress while simultaneously inhibiting FOXO3-induced cell death (42). Consequently, SIRT1 may extend organismal lifespan by shifting P53 and/or FOXO-dependent responses from programmed cell death towards stress resilience.NF-κB is a critical regulator of gene expression related to cellular survival. SIRT1 physically associates with the RELA/p65 subunit of NF-κB and suppresses its transcriptional activity through deacetylation at lysine 310. The loss of NF κB-regulated gene expression makes cells more susceptible to TNFα-induced apoptosis (43). SIRT1 has dual roles in controlling cell survival: it can protect cells from apoptotic signals initiated by p53 or FOXO, yet its activity can also enhance apoptosis in response to TNFα. This dual role underscores the complex and context-dependent nature of SIRT1’s involvement in apoptosis regulation.

### 2.2 SIRT1 and inflammation

Inflammation is a complex immune response initiated by the body in response to injury, infection, or stimulation. As a vital part of the immune system, inflammation aids in pathogen clearance, tissue repair, and the restoration of tissue function (44). Symptoms typically associated with inflammatory reactions (45) include redness, swelling, fever, pain, and loss of function. Inflammatory diseases, a particular category of illnesses, are caused by chronic inflammation. These conditions are characterized by protracted, persistent, or repeated inflammatory responses that can lead to tissue damage and dysfunction, impacting multiple organs and systems. Common inflammatory diseases include rheumatoid arthritis (RA) (46), inflammatory bowel disease (IBD) (47), psoriasis (48), systemic lupus erythematosus (SLE) (49), ankylosing spondylitis (AS) (50), and asthma (51). Changes in the expression and activity of SIRT1 can disrupt normal cellular processes, thereby contributing to the onset and progression of these diseases.

SIRT1 exhibits substantial anti-inflammatory properties and can mitigate inflammatory damage by inhibiting key pro-inflammatory pathways, such as NF-κB (52) and PARP1 (53). It exerts its anti-inflammatory effect by deacetylating the p65 subunit of NF-κB at the lysine 310 residue (43). This molecular modification effectively suppresses NF-κB activity and curbs the production of downstream inflammatory cytokines. For example, research has indicated that mice lacking SIRT1, when exposed to particulate matter, suffer from heightened NF-κB activation and more severe lung inflammation compared to their wild-type counterparts. In line with these findings, overexpression of SIRT1 has been seen to dampen NF-κB signaling and alleviate the aggressive behavior and inflammatory responses of fibroblast-like synoviocytes in patients with rheumatoid arthritis. As such, SIRT1 has emerged as a promising therapeutic target for addressing aging-related disorders associated with chronic inflammation.

**Figure 1:**
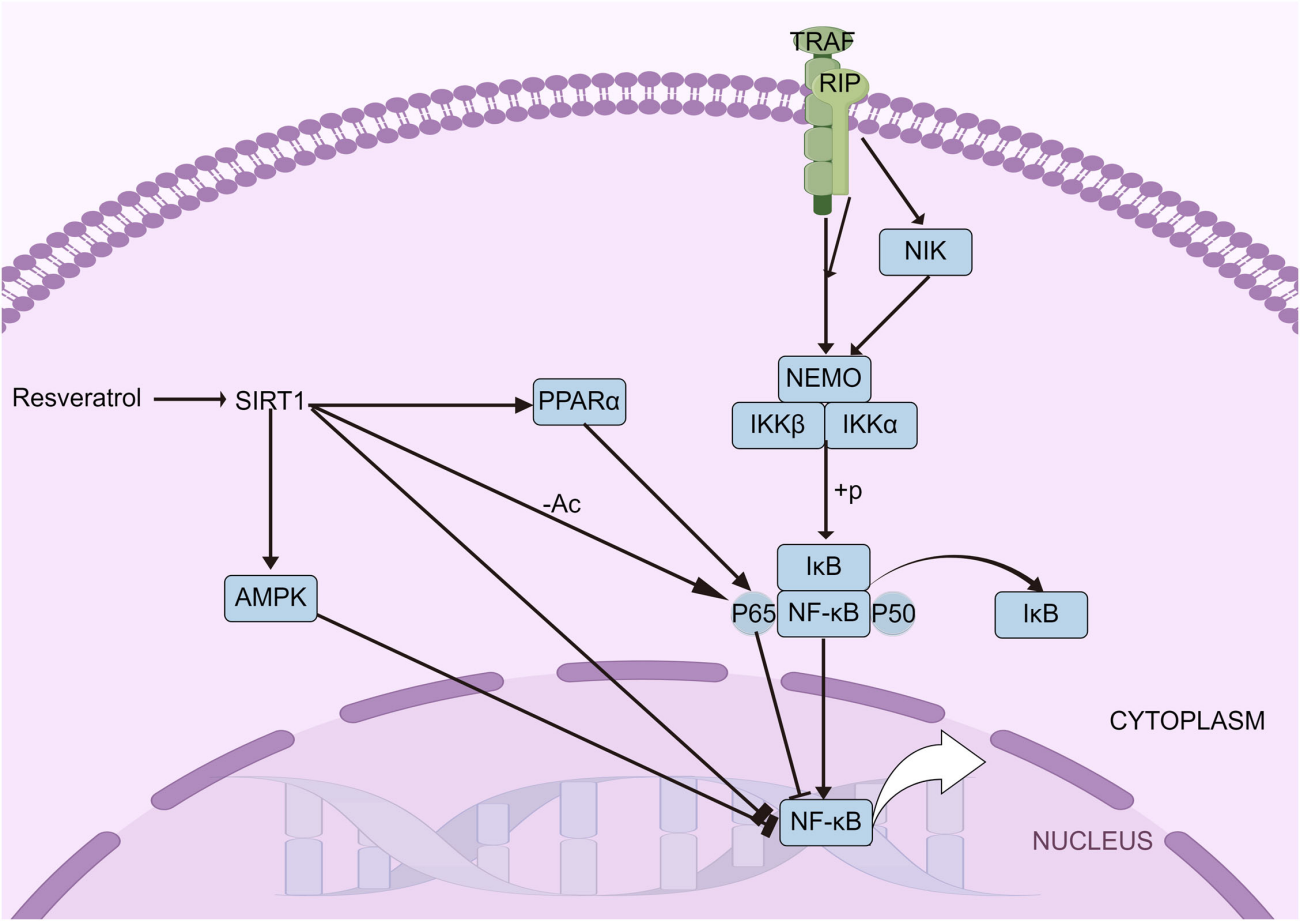
NF-κB signaling pathways. The NF-κB pathway, vital in sepsis-related inflammation, involves subunits like NF-κB1, NF-κB2, and P65. Normally inactive in the cytoplasm, NF-κB is activated by IκB degradation, moving to the nucleus to trigger immune genes. SIRT1 inhibits NF-κB by deacetylating P65, with resveratrol reducing P65’s nuclear presence and DNA binding.

The NF-κB signaling pathway, crucial in the inflammatory response during sepsis, is one of the most thoroughly studied pathways in inflammation research. The NF-κB complex is composed of various subunits (54) including NF-κB1 (P105 and P50), NF-κB2 (P100 and P52), P65, RELB, and c-REL. Under normal, unstimulated conditions, NF-κB remains inactive in the cytoplasm, bound to members of the inhibitor of κB (IκB) family. However, when stimulated by an inflammatory trigger, IκB is phosphorylated and degraded by the IκB kinase (IKK), enabling NF-κB to migrate to the nucleus and activate gene transcription essential for immune and inflammatory responses. SIRT1 has been found to directly suppress inflammatory gene expression by targeting NF-κB. Studies have shown that SIRT1 deacetylates the P65 subunit of NF-κB, thereby inhibiting its activity (43). SIRT1 influences both the nuclear transport of NF-κB and its DNA binding capacity. Research has indicated that resveratrol, a known SIRT1 activator, inhibits the accumulation of P65 in the nucleus and reduces its DNA binding ability (55). Inflammatory stimuli prompt SIRT1 to accumulate at the transcriptional regulatory regions of NF-κB target genes. Studies have also shown that chronic exposure to LPS leads to the accumulation of SIRT1 at the promoters of inflammatory cytokines, resulting in the deacetylation of P65 (56). The impacts of SIRT1 on the activation of various molecular targets within the NF-κB signaling pathway during the inflammatory response are significant (**Figure 1**).

SIRT1 indirectly moderates NF-κB signaling by influencing the expression of mediator proteins such as AMPK and PPARs. The interaction between SIRT1 and AMPK significantly affects the inflammatory response. Acting as an inhibitor of NF-κB, AMPK can be activated by SIRT1, which leads to an indirect reduction in NF-κB activity (52). Research has shown that overexpression of SIRT1 enhances the interaction between PPARα and P65, thereby inhibiting NF-κB activation and reducing MCP-1 transcription (57). The relationship between SIRT1 and NF-κB is thus bidirectional. In clinical settings, however, the serum levels of SIRT1 in patients exhibit varying patterns depending on the condition. Patients with acute pancreatitis and rheumatoid arthritis show increased levels of serum SIRT1 (23, 58), whereas those with sepsis and chronic obstructive pulmonary disease exhibit decreased levels (26, 28). This discrepancy highlights the complex and still unclear role of SIRT1 in inflammatory diseases.

## 3 Methods

### 3.1 Publication search strategy

Given the ambiguous role of SIRT1 in inflammatory diseases, we embarked on a meta-analysis of all studies pertinent to SIRT1 and inflammation to dissect the critical aspects of SIRT1 immunobiology relevant to inflammation. This comprehensive review utilized the PubMed, Cochrane Library, and Web of Science databases to collate studies on Serum SIRT1 levels in patients with inflammatory conditions, spanning from the databases’ inception through March 2024, without setting a lower date limit. We imposed no language restrictions and meticulously examined the reference lists of all identified articles to unearth additional studies of potential interest. This systematic review and meta-analysis were conducted in strict adherence to the Preferred Reporting Items for Systematic Reviews and Meta-Analyses (PRISMA) guidelines (59). Our primary search deployed terms such as “Sirtuin 1,” “Sirt1,” and “Silent Mating Type Information Regulation 2 Homolog 1,” in conjunction with specific terminology for various inflammatory diseases. These diseases included rheumatoid arthritis (and rheumatic fever), systemic lupus erythematosus, systemic sclerosis (diffuse scleroderma), localized scleroderma, gout, inflammatory myopathy (including myositides and dermatopolymyositis), ophthalmia (endophthalmitides), inflammatory bowel disease (Crohn’s disease and ulcerative colitis), asthma, rhinitis (allergic rhinitides), dermatitides, nephritis, hepatitis, endocarditis, encephalitis, pancreatitis, thyroiditis (Hashimoto’s disease), enteritis (appendicitis), pneumonia, osteomyelitis, bronchitis, chronic obstructive pulmonary disease, gastritis, pharyngitis, prostatitis, vaginitis, otitis media, ankylosing spondylitis, periodontitis, systemic inflammatory response syndrome (sepsis), optic neuritis, cholangitis, psoriasis, psoriatic arthritis, atherosclerosis, tonsillitis, cellulitis, and more. The detailed search strategy employed for the PubMed database can be found in **Supplementary Appendix 1**.

### 3.2 Inclusion and exclusion criteria

The inclusion criteria for studies in this meta-analysis were as follows: [1] Inclusion of patients diagnosed with inflammatory conditions along with a control group for comparison. [2] An observational study design. [3] Measurement of SIRT1 levels via laboratory methods, specifically using ELISA for detection. [4] The study must be published in English. [5] A minimum sample size of 10 individuals per study group is required. Studies were excluded based on the following criteria: [1] Lack of necessary data for analysis. [2] Research involving animal subjects. [3] Submissions categorized as letters, comments, correspondence, editorials, or reviews rather than original research. [4] Studies where relevant outcomes were not reported or could not be derived from the published data. [5] Publications representing duplicate data sets or patient cohorts. [6] Articles not composed in English. [7] Studies focusing on non-inflammatory conditions.

### 3.3 Data extraction

Two investigators were tasked with the literature compilation process, each independently extracting data from the selected studies and subsequently cross-verifying their findings. In cases where discrepancies arose, a third investigator was consulted to mediate and reach a consensus. The data extracted encompassed several key elements: [1] Fundamental details of the studies, such as the title, authors, publication date, sample sizes for both control and experimental groups, and the source of the literature. [2] Levels of SIRT1, reported as means ± standard deviations or medians [interquartile ranges], along with the specific types of inflammatory diseases under investigation. [3] Details regarding the origin of the samples and the methodologies employed for testing. [4] The primary and secondary outcome measures of the studies. To assess the quality of the non-randomized studies included in the meta-analysis, the Newcastle-Ottawa Scale (NOS) (60) was employed, as proposed by Wells et al. (61). The NOS evaluates studies across three main domains: the selection of patients, the comparability of the case/exposure groups with the controls, and the assessment of exposure. Each criterion within the selection and outcome categories can earn a study a maximum of one star. The quality of the studies was thus rated based on their NOS scores, which ranged from 0 to 9. Studies achieving a score of 6 or higher were considered to be of high quality.

### 3.4 Statistical methods

In this study, data management and visualization were performed using STATA 12.0 software. Forest plots were generated to depict categorical data using binary variable meta-analysis, while continuous variables (such as sample size, mean, and standard deviation) were analyzed using continuous variable meta-analysis. Continuous outcome variables were presented as weighted mean differences (WMDs) and the corresponding 95% confidence intervals (CIs) acted as summary statistics. The statistical significance between two groups of continuous data was determined by the overall difference, indicated by the z-value and p-value. Cochran’s *Q* statistic and the *I^2^* statistic were utilized to assess statistical heterogeneity (62, 63). Significant heterogeneity was inferred if the *P* value was less than 0.05. The *I^2^* value was used to gauge the extent of heterogeneity, with values of 25%, 50%, and 75% indicating low, moderate, and high levels of heterogeneity, respectively (62, 64). In cases where *P*> 0.05 and *I^2^* < 25%, suggesting negligible heterogeneity, a fixed effects model was used to pool the overall results. Conversely, a random effects model was employed in the presence of significant heterogeneity (65). The random-effects model tends to be more robust when there is unexplained heterogeneity, as it does not assume that all studies are estimating the same effect size. Due to its assumption of additional variability, the random-effects model typically produces wider confidence intervals, which provide a more conservative estimate when heterogeneity is present. Publication bias for the included studies was evaluated using funnel plots for categorical data (66) and Begg’s and Egger’s tests (67, 68) for continuous data. A *P/p*-value less than 0.05 was considered indicative of statistically significant differences.

## 4 Results

### 4.1 Study characteristics

**Figure 2:**
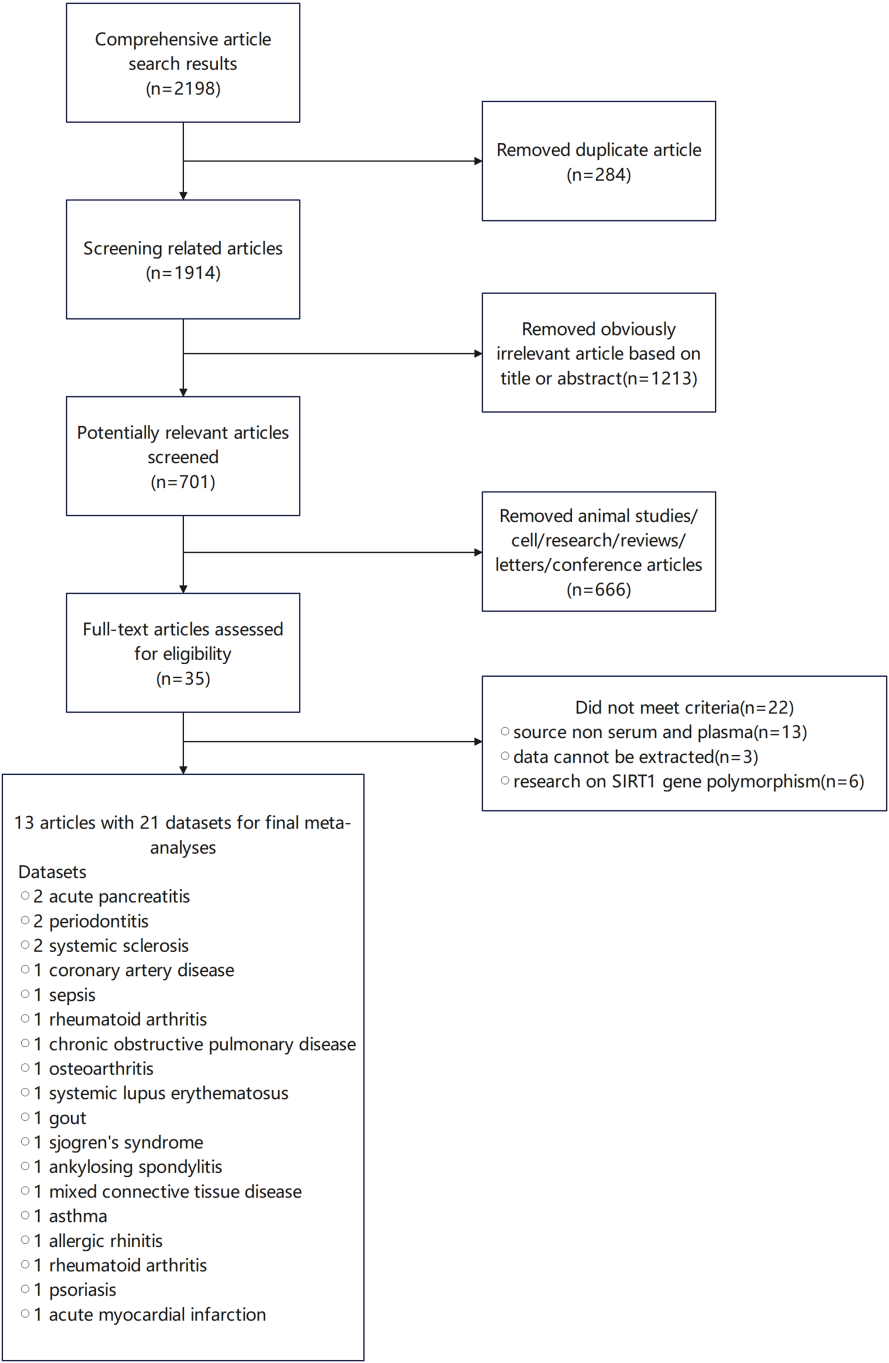
Study inclusion flow diagram in the systematic review and meta-analysis according to the preferred reporting items for systematic reviews and meta-analysis (PRISMA).

Initially, 2198 studies were screened, from which 2163 were excluded after a review of titles and abstracts due to duplication, irrelevance, or non-original research forms such as reviews and letters. This left 35 studies for detailed review. Ultimately, 13 studies, encompassing 21 data groups with a total of 2,028 participants, were deemed suitable for inclusion in our pooled analyses (23–32, 58, 69, 70). All included studies assessed SIRT1 expression using enzyme-linked immunosorbent assay (ELISA). The specifics of these studies are provided in **Supplementary Appendix 2**, (**Figure 2**). 

### 4.2 Meta-analysis

**Figure 3:**
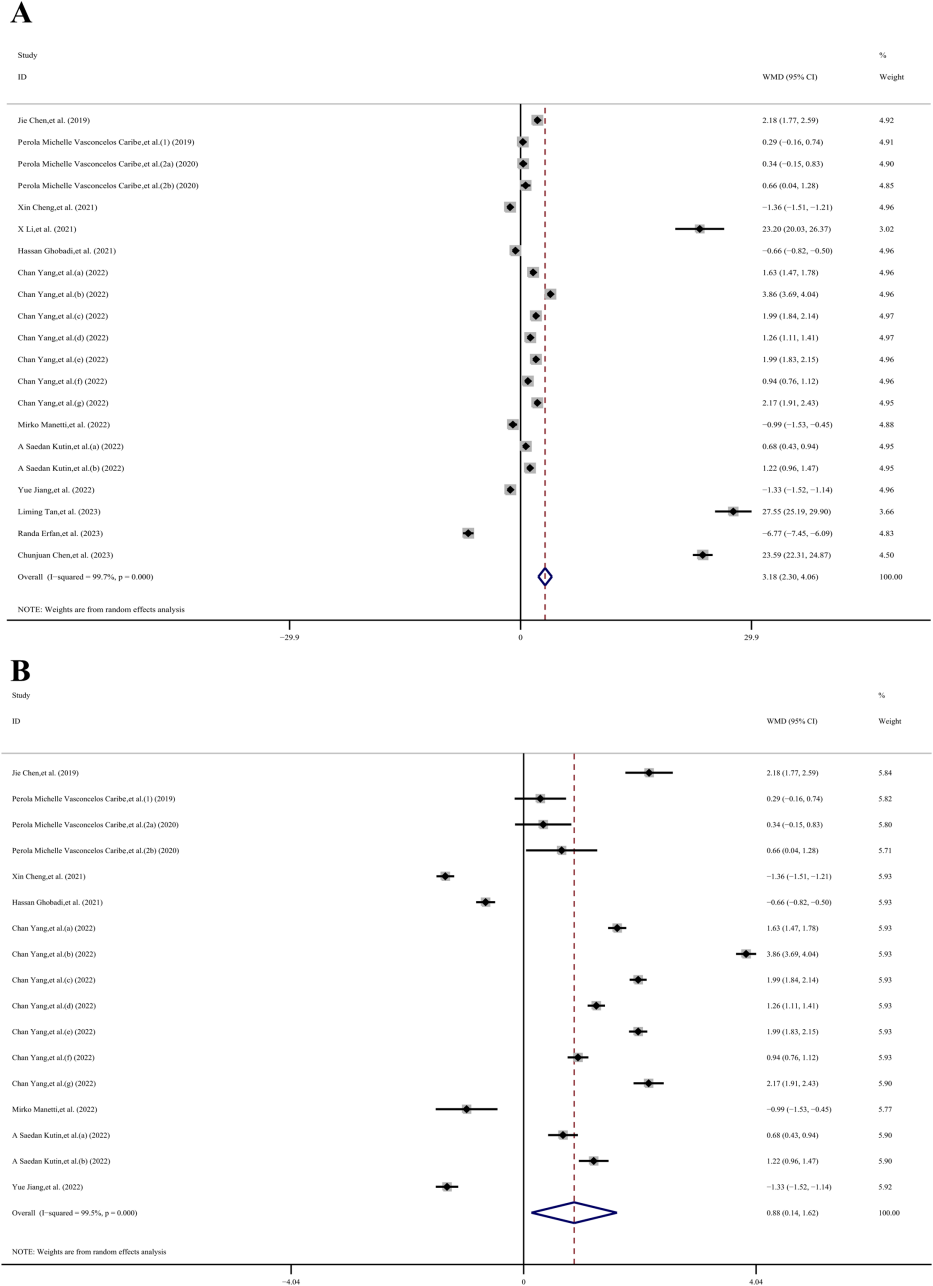
The forest plot shows the results of the meta-analysis of SIRT1 and inflammatory diseases. **(A)** Meta-analysis of concertation of SIRT1 and inflammatory diseases in 21 datasets; **(B)** 17 datasets after 4 datasets which reported high SIRT1 base level (>10ng/ml) were removed.

The meta-analysis incorporated a total of 5 case-control datasets and 16 cross-sectional datasets, all of which provided the mean ± SD of SIRT1 concentration in individuals with and without inflammatory diseases. Among these studies, 9 utilized plasma samples while 12 used serum samples. The meta-analysis revealed that the SIRT1 concentration in the case group was 3.18 ng/mL higher than that in the control group (WMD, 3.18 ng/mL;95% CI 2.30, 4.06 ng/mL; *p*< 0.001; *I^2^ =* 99.7%), as per a random effects model. This demonstrated substantial heterogeneity. However, four studies (27, 58, 69, 70) presented baseline SIRT1 levels greater than 10 ng/mL for the control group. To enhance the relevance of our meta-analysis results, we sequentially removed these four studies. Following this adjustment, the meta-analysis indicated that the SIRT1 concentration in the case group was 0.88 ng/mL higher than that in the control group (WMD, 0.88 ng/mL;95% CI 0.14,1.62 ng/mL; *p*< 0.001; *I^2^ =* 99.5%), using a random effects model. Even after this adjustment, substantial heterogeneity was observed, suggesting that other factors or study-specific characteristics might be influencing the results (**Figure 3**).

### 4.3 Subgroup analysis

Given the high heterogeneity observed, we conducted a subgroup analysis to explore potential sources of variability. This analysis was stratified by age (young, middle-aged, elderly), region (eastern Asia, western Asia), sample type (serum, plasma), and type of immune system disease (other inflammatory diseases, infectious diseases, chronic degenerative diseases, autoimmune disease, allergic diseases), as well as disease category (digestive system diseases, oral and maxillofacial joint diseases, respiratory disease, musculoskeletal disorders, skin and connective tissue diseases). Due to the unavailability of age data in 6 datasets, we were able to include only 11 datasets (23–26, 28–32) for the age subgroup analysis. The overall result across these datasets did not show statistical significance (WMD, −0.45 ng/mL; 95% CI −0.77, 1.66 ng/mL; *P*= 0.473; *I^2^ =* 99.6%). However, within the middle-aged group, the SIRT1 concentration in the case group was 0.85 ng/mL lower than in the control group (WMD, −0.85 ng/mL; 95% CI −1.47, −0.22 ng/mL; *P*= 0.008; *I^2^ =* 95.4%). In contrast, there was no statistical significance observed in the other age groups: the young-aged group showed a WMD of 1.92 ng/mL (95% CI −0.20, 4.04 ng/mL; *p*= 1.78; *I^2^ =* 99.6%), and the elderly-aged group had a WMD of 0.29 ng/mL (95% CI −0.86, 1.45 ng/mL; *p*= 0.50; *I^2^ =* 97.8%).

Due to the unavailability of region data in one dataset, we included 16 datasets (23–26, 28, 29, 31, 32) in our region subgroup analysis. Indicate that the overall effect was statistically significant (WMD, 0.99 ng/mL; 95% CI 0.23, 1.76 ng/mL; *P*= 0.011; *I^2^ =* 99.6%). Specifically, in the Eastern Asia subgroup, the SIRT1 concentration in the case group was 1.33 ng/mL higher than in the control group (WMD, 1.33 ng/mL; 95% CI −0.32, 2.34 ng/mL; *P*= 0.01; *I^2^ =* 99.7%). However, in the Western Asia group, there was no statistically significant difference (WMD, 0.42 ng/mL; 95% CI −0.32, 1.16 ng/mL; *p*= 0.27; *I^2^ =* 97.2%).

The sample subgroup analysis (23–26, 28–32), revealed that in the plasma sample group, the SIRT1 concentration in the case group was 1.98 ng/mL higher than in the control group (WMD, 1.98 ng/mL; 95% CI 1.30, 2.66 ng/mL; *P*< 0.001; *I^2^ =* 99.1%). However, in the serum sample group, there was no statistically significant difference (WMD, 0.10 ng/mL; 95% CI −0.62, 0.81 ng/mL; *p*= 0.792; *I^2^ =* 98.6%).

The results of the immune system disease subgroup analysis (23–26, 28, 29, 31, 32), revealed that in the autoimmune disease group, the SIRT1 concentration in the case group was 1.46 ng/mL higher than in the control group (WMD, 1.46 ng/mL; 95% CI 0.16, 2.77 ng/mL; *P*= 0.027; *I^2^ =* 99.5%). Similarly, in the allergic diseases group, the SIRT1 concentration in the case group was 0.95 ng/mL higher than that in the control group (WMD, 0.95 ng/mL; 95% CI 0.43, 1.47 ng/mL; *P*< 0.001; *I^2^ =* 87.9%). However, there was no statistically significant difference in the other disease groups. Specifically, the other inflammatory diseases group showed a WMD of 0.94 ng/mL (95% CI −1.50, 3.39 ng/mL; *p*= 0.45; *I^2^ =* 99.7%), the infectious diseases group had a WMD of -0.26 ng/mL (95% CI −1.57, 1.05 ng/mL; *p*= 0.699; *I^2^ =* 97.5%), and the chronic degenerative diseases group exhibited a WMD of 0.91 ng/mL (95% CI −0.46, 2.27 ng/mL; *p*= 0.193; *I^2^ =* 99.5%).

**Figure 4:**
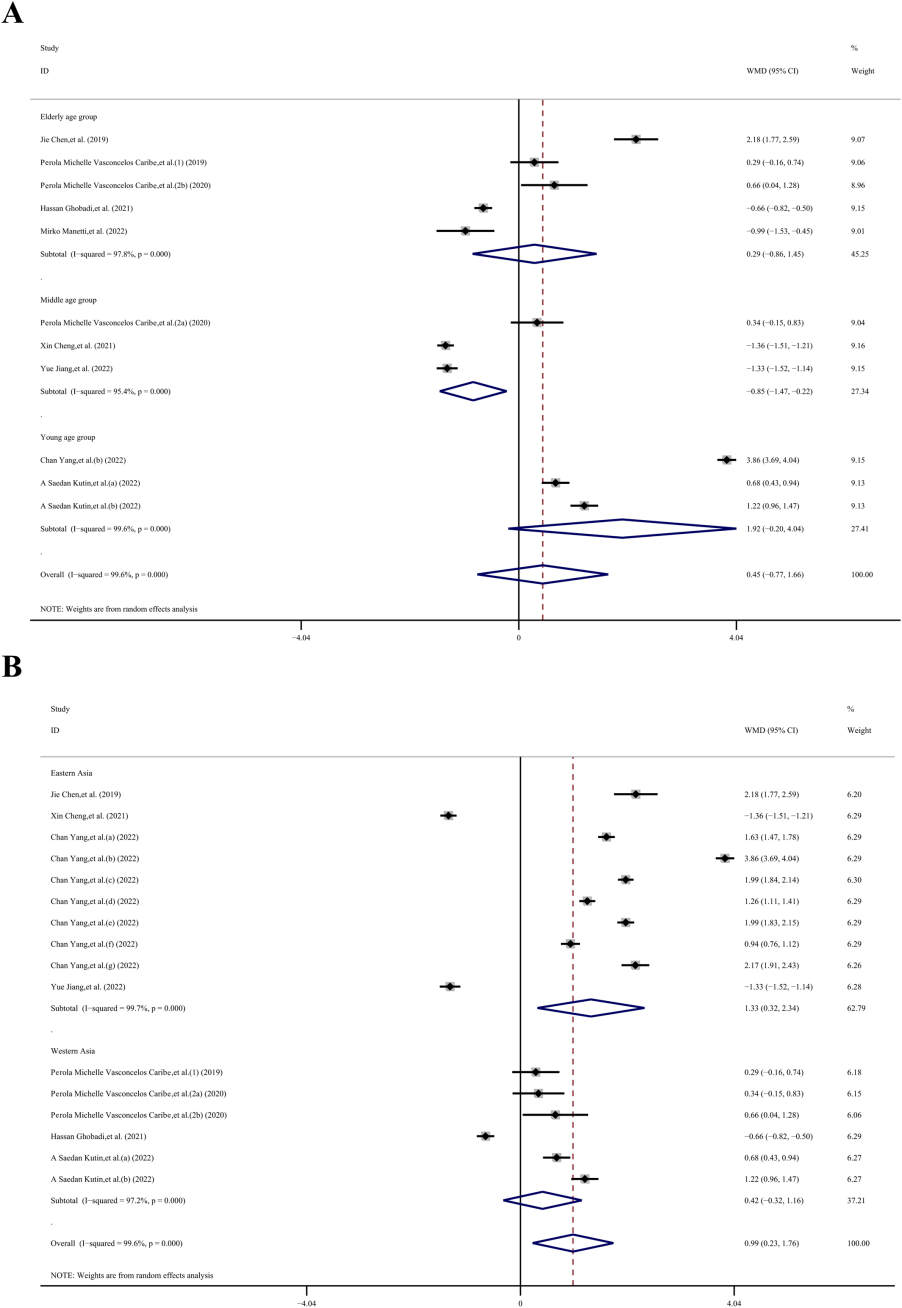
Subgroup analysis stratified by **(A)** age (young, middle-aged, elderly); **(B)** region (eastern Asia, western Asia).

**Figure 5:**
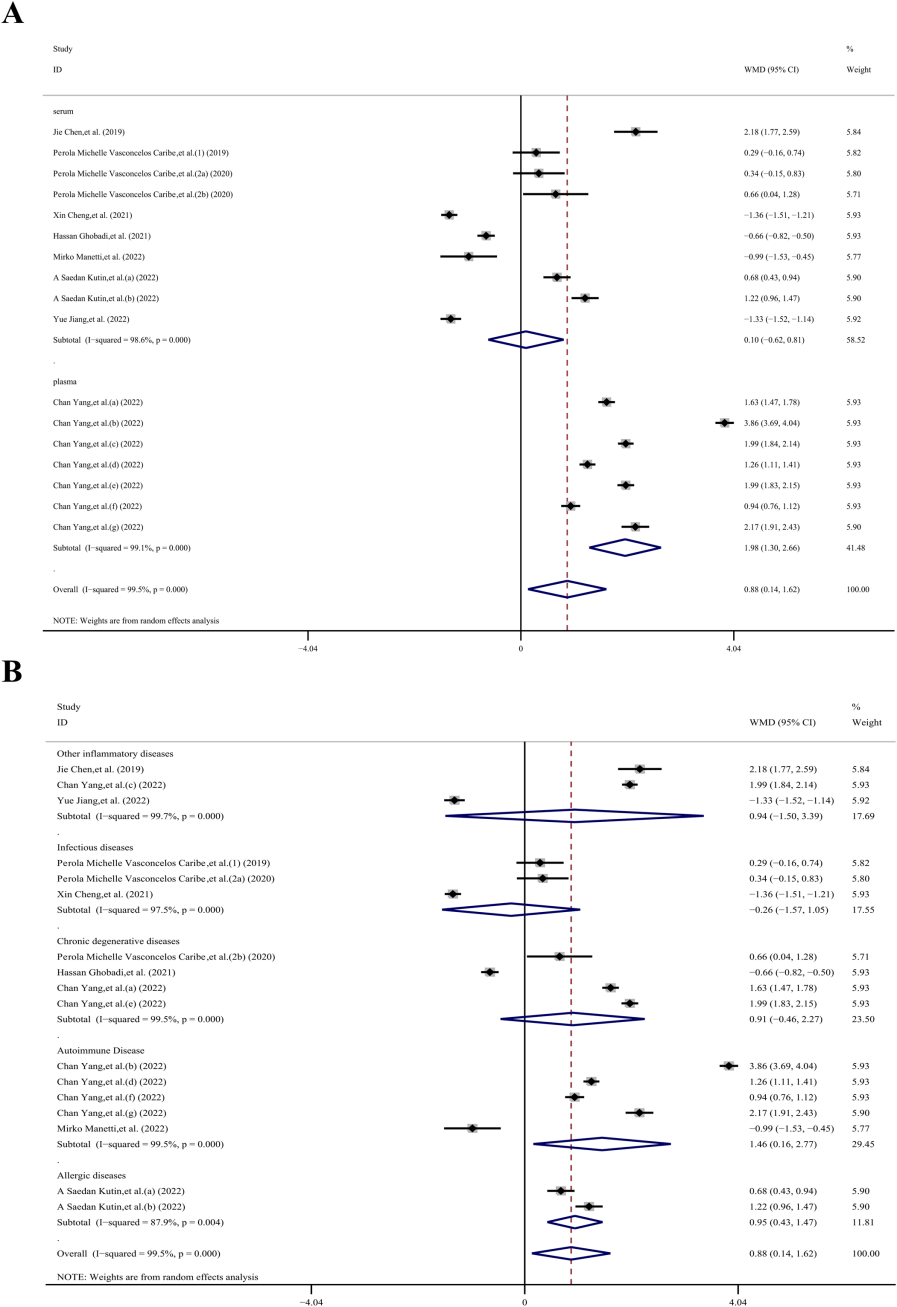
Subgroup analysis stratified by **(A)** sample type (serum, plasma); **(B)** immune system disease (other inflammatory diseases, infectious diseases, chronic degenerative diseases, autoimmune disease, allergic diseases).

**Figure 6:**
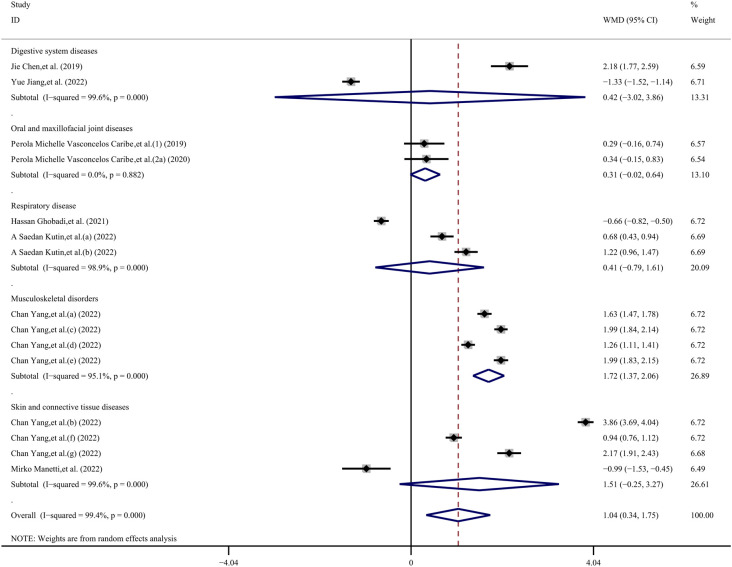
Subgroup analysis stratified by disease category (digestive system diseases, oral and maxillofacial joint diseases, respiratory disease, musculoskeletal disorders, skin and connective tissue diseases).

Due to the inability to extract disease category data from 2 datasets, we included a total of 15 datasets in our analysis (23–25, 28–32). The results of the disease category subgroup analysis, indicate that the overall result was statistically significant (WMD, 1.04 ng/mL; 95% CI 0.34, 1.75 ng/mL; *P*= 0.004; *I^2^ =* 99.4%). Specifically, in the musculoskeletal disorders group, the SIRT1 concentration in the case group was 1.72 ng/mL higher than in the control group (WMD, 1.72 ng/mL; 95% CI 1.37, 2.06 ng/mL; *P*< 0.001; *I^2^ =* 95.1%). However, there was no statistically significant difference in other disease category groups. The digestive system diseases group showed a WMD of 0.42 ng/mL (95% CI −3.02, 3.86 ng/mL; *p*= 0.811; *I^2^ =* 99.6%), the oral and maxillofacial joint diseases group had a WMD of 0.31 ng/mL (95% CI −0.02, 0.64 ng/mL; *p*= 0.063; *I^2^ =* 0%), the respiratory disease group exhibited a WMD of 0.41 ng/mL (95% CI −0.79, 1.61 ng/mL; *p*= 0.504; *I^2^ =* 98.9%), and the skin and connective tissue diseases group presented a WMD of 1.51 ng/mL (95% CI −0.25, 3.27 ng/mL; *p*= 0.093; *I^2^ =* 99.6%) (**Figures 4**–**6**).

### 4.4 Sensitivity analysis

**Figure 7:**
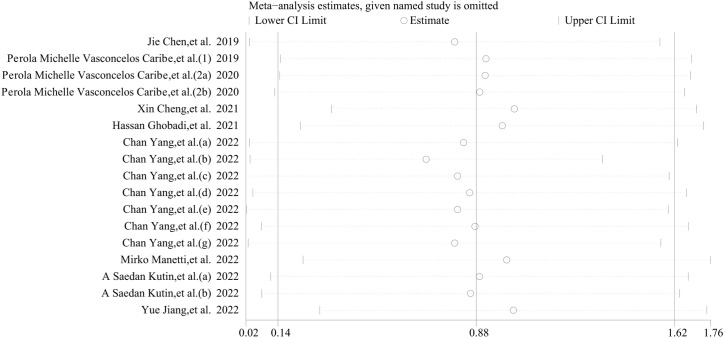
Sensitivity analyses of the studies. The analysis demonstrated that individual studies exerted minimal influence on the overall outcomes, thereby corroborating the stability and credibility of our findings.

Stata 12.0 software was employed to conduct a sensitivity analysis aimed at assessing the stability of our results. The analysis revealed that individual studies had minimal influence on the final outcomes, thereby validating the stability and credibility of our findings (**Figure 7**). Given the high heterogeneity observed in this study, we hypothesize that this may be attributable to the cross-sectional nature of all the data we extracted.

### 4.5 Publication bias

**Figure 8:**
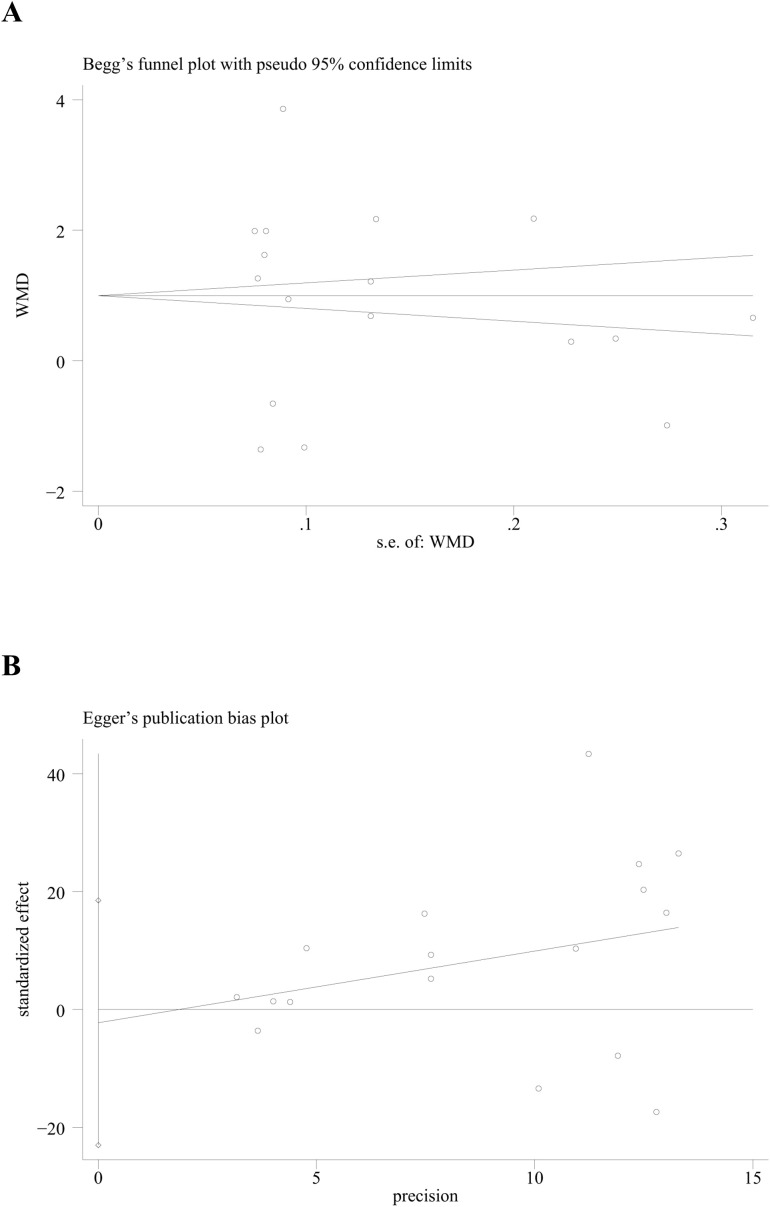
Publication bias analyses. **(A)** Begg’s test; **(B)** Egger’s test for publication bias.

Both Begg’s test and Egger’s test were utilized to assess the presence of publication bias. The results from these tests (*p* for Begg’s test = 0.266; *p* for Egger’s test = 0.820) indicated that no publication bias was present across all subgroups, as all the p-values were greater than 0.05 (**Figure 8**).

## 5 Discussion

In this meta-analysis, we examined 13 studies comprising 21 cross-sectional datasets with a total of 1,219 patients suffering from inflammatory diseases to explore the role of SIRT1 in inflammation. Our findings indicate that SIRT1 is upregulated in cases of inflammatory diseases, suggesting its potential role as a regulator in the inflammatory process. Within the studies included, sixteen datasets reported an increase in SIRT1 levels in the inflammatory group, whereas five datasets indicated a decrease. This discrepancy suggests that a decrease in SIRT1 levels may be significantly associated with the prevalence of various inflammatory diseases (71). Upregulation of SIRT1 has been linked to the suppression of neuroinflammation (72), highlighting how the body may counteract the progression of inflammation by increasing SIRT1 levels. SIRT1 is predominantly located in the nucleus (73), but recent studies suggest it also localizes to the mitochondria. The relationship between SIRT1 and inflammation is complex, with various studies yielding differing conclusions. Some research suggests that SIRT1 exhibits anti-inflammatory properties by inhibiting the activation of NF-κB, a critical regulator of the inflammatory response. The deacetylation activity of SIRT1 may attenuate NF-κB activity, thereby mitigating inflammation. Conversely, SIRT1 may in some instances amplify the inflammatory response by regulating the inflammatory response’s negative feedback mechanism (52). Furthermore, the role of nuclear autophagy in cellular aging and the aging process has been highlighted, with findings that SIRT1, a key metabolic and aging regulator, is selectively degraded by autophagy in aging across several tissues, including those of the hematopoietic and immune systems. Stabilizing SIRT1 protein emerges as a novel approach to promoting healthy and effective aging. Additionally, the study unveiled that nuclear autophagy fosters senescence characteristics such as cell cycle arrest and the pro-inflammatory program, both of which are crucial for tumor suppression (74).

Based on the findings of our meta-analysis, we hypothesize that the progression of inflammation disrupts bodily homeostasis, which in turn affects the upregulation of SIRT1, aiming to mitigate inflammation. This hypothesis is supported by the results of our meta-analysis. The fundamental principle of a meta-analysis is the assumption of a common truth underlying all conceptually similar scientific studies, despite each study having its own degree of error. Therefore, the objective of a meta-analysis is to employ statistical methods to derive a pooled estimate that closely approximates this common truth, taking into account the perceived errors. This approach is advantageous because it consolidates information from multiple sources, resulting in greater statistical power and more robust point estimates than could be achieved by any single study.

Our meta-analysis reveals that serum levels of SIRT1 are elevated during inflammatory conditions, suggesting its significant role in the disease’s pathophysiological processes and potential as a therapeutic target. SIRT1’s ability to modulate inflammatory pathways positions it as a critical player in these mechanisms. Unfortunately, we were unable to find publications containing longitudinal studies on this subject to incorporate into our analysis, which might have revealed more detailed mechanisms. Nevertheless, our findings indicate that SIRT1 could serve as a valuable biomarker for disease progression and treatment response. Monitoring SIRT1 levels could enable clinicians to track the disease course more accurately and make timely adjustments to treatment protocols. Additionally, the responsiveness of SIRT1 levels to therapeutic interventions may serve as an indicator of treatment efficacy, facilitating more personalized and effective management of inflammatory diseases. Future research should focus on validating SIRT1 as a reliable biomarker through longitudinal studies and clinical trials. Understanding the precise mechanisms by which SIRT1 influences inflammatory pathways will be crucial for developing novel therapeutic strategies. Such insights could potentially improve outcomes for patients with inflammatory diseases, positioning SIRT1 as a central focus in both diagnostic and therapeutic contexts.

The age subgroup analysis revealed a negative correlation between inflammation and SIRT1 levels among middle-aged individuals, while no such effect was detected in younger and elderly subjects, suggesting variations across different age groups. However, the limited age range of the patients, which spans from 40 to 60 years old and predominantly includes middle-aged to elderly individuals, precludes the drawing of definitive conclusions. In the regional subgroup analysis, a significant positive correlation between inflammation and SIRT1 levels was observed in participants from Eastern Asia. It is important to note that this subgroup included 7 datasets from a single study, which could potentially introduce bias. Similarly, the sample subgroup analysis indicated a significant positive correlation between inflammation and SIRT1 levels in plasma samples, with seven datasets also originating from a single study, posing a high risk of bias. The subgroup analysis focusing on immune system diseases demonstrated a significant positive correlation between inflammation and SIRT1 levels in individuals with autoimmune diseases. Additionally, the disease category subgroup analysis showed a significant positive correlation between inflammation and SIRT1 levels in subjects suffering from musculoskeletal disorders. Nonetheless, the inability to establish a standardized timeline for the progression of inflammatory diseases may introduce variability and potentially impact the results of our analysis.

This study faced several limitations. Firstly, all included studies were observational, which inherently provides a lower level of evidence. Additionally, the meta-analysis was based on a restricted number of studies that met our inclusion and exclusion criteria, and significant heterogeneity was observed among them, likely due to the presence of numerous confounding variables. For instance, we exclusively utilized data on SIRT1 levels in blood, quantified through ELISA. Consequently, it is necessary to conduct a broader range of studies to assess the impact of other study characteristics on the outcomes, such as SIRT1 gene expression (measured via qPCR or RT-PCR) and SIRT1 protein content (measured via Western blot). Begg’s and Egger’s tests indicated that there was no evidence of publication bias in the meta-analysis conducted for this study. However, the sample sizes of the included studies varied considerably, and the inflammatory diseases studied did not adhere to a standardized timeline for progression. This lack of uniformity in baseline conditions across studies may have influenced our results.

## 6 Conclusions

Our meta-analysis revealed a positive correlation between SIRT1 levels and inflammation, indicating that more severe inflammatory conditions are associated with higher levels of SIRT1. However, these findings should be interpreted with caution, as all the studies included were cross-sectional in nature. Given this limitation, additional data and fundamental research are required to further substantiate the relationship between SIRT1 and inflammation. Overall, these insights could contribute to a better understanding of the immunobiological roles of SIRT1 in inflammatory diseases and potentially aid in the development of new therapeutic strategies aimed at reducing the burden of these diseases.
