# Supplementary material for: The relationship between SIRT1 and inflammation: a systematic review and meta-analysis
Source: Front Immunol. 2024 Nov 29;15:1465849. doi: 10.3389/fimmu.2024.1465849 (PMC11638041; doi:10.3389/fimmu.2024.1465849)
Supplement: Supplementary file 1 [file Table1.docx]

Supplementary Material

# Supplementary Table1

Supplementary appendix1: Search strategy performed at PubMed, at March 20, 2024

| Search | PubMed search strategy | Results |
| --- | --- | --- |
| 1 | ((Sirtuin 1) **OR** (Sirt1)) **OR** (Silent Mating Type Information Regulation 2 Homolog 1) | 14826 |
| 2 | ((Rheumatoid Arthritis) **OR** (Rheumatic Fever)) **OR** (Arthritis) | 416740 |
| 3 | (Systemic Lupus Erythematosus) | 85772 |
| 4 | (Systemic Sclerosis) **OR** (Diffuse Scleroderma)  (Localized Scleroderma) | 35536  5485 |
| 5 | (Gouts) | 23091 |
| 6 | ((Inflammatory Myopathy) **OR** (Myositides)) **OR** (Dermatopolymyositis) | 31168 |
| 7 | (Endophthalmitides) **OR** (Ophthalmia) | 13945 |
| 8 | (Inflammatory Bowel Disease) **OR** (Crohn's Enteritis)  (Inflammatory Bowel Disease) **OR** (Ulcerative Colitis) | 141328  139479 |
| 9 | (Asthmas) | 222722 |
| 10 | (Rhinitis) **OR** (Allergic Rhinitides) | 52403 |
| 11 | (Dermatitides) | 143742 |
| 12 | (Nephritis) | 91291 |
| 13 | (Hepatitis) | 294566 |
| 14 | (Endocarditis) | 46351 |
| 15 | (Encephalitis) | 78774 |
| 16 | (Pancreatitis) | 419107 |
| 17 | (Thyroiditis) **OR** (Hashimoto Disease) | 270542 |
| 18 | (Enteritis) **OR** (Appendicitis) | 103660 |
| 19 | (Pneumonia) | 514659 |
| 20 | (Osteomyelitis) | 35674 |
| 21 | (Bronchitis) | 44801 |
| 22 | (Chronic Obstructive Lung Disease) | 104370 |
| 23 | (Gastritis) | 33749 |
| 24 | (Pharyngitis) | 20059 |
| 25 | (Prostatitis) | 277329 |
| 26 | (Vaginitis) | 165773 |
| 27 | (Otitis Media) | 33864 |
| 28 | (Ankylosing Spondylitis) | 22574 |
| 29 | (Periodontitis) | 130749 |
| 30 | (Systemic Inflammatory Response Syndrome) **OR** (Sepsis) | 231620 |
| 31 | (Optic Neuritis) | 13716 |
| 32 | (Cholangitis) | 22425 |
| 33 | (Psoriasis) | 65713 |
| 34 | (Psoriatic Arthritis) | 14576 |
| 35 | (Atherosclerosis) | 178445 |
| 36 | (Tonsillitis) | 28481 |
| 37 | (Cellulitis) | 15657 |
| 38 | 1 **AND** 2 | 269 |
| 39 | 1 **AND** 3 | 29 |
| 40 | 1 **AND** 4 | 12 |
| 41 | 1 **AND** 5 | 17 |
| 42 | 1 **AND** 6 | 6 |
| 43 | 1 **AND** 7 | 0 |
| 44 | 1 **AND** 8 | 71 |
| 45 | 1 **AND** 9 | 63 |
| 46 | 1 **AND** 10 | 15 |
| 47 | 1 **AND** 11 | 18 |
| 48 | 1 **AND** 12 | 30 |
| 49 | 1 **AND** 13 | 98 |
| 50 | 1 **AND** 14 | 0 |
| 51 | 1 **AND** 15 | 15 |
| 52 | 1 **AND** 16 | 372 |
| 53 | 1 **AND** 17 | 118 |
| 54 | 1 **AND** 18 | 15 |
| 55 | 1 **AND** 19 | 71 |
| 56 | 1 **AND** 20 | 2 |
| 57 | 1 **AND** 21 | 4 |
| 58 | 1 **AND** 22 | 133 |
| 59 | 1 **AND** 23 | 4 |
| 60 | 1 **AND** 24 | 1 |
| 61 | 1 **AND** 25 | 140 |
| 62 | 1 **AND** 26 | 10 |
| 63 | 1 **AND** 27 | 1 |
| 64 | 1 **AND** 28 | 3 |
| 65 | 1 **AND** 29 | 60 |
| 66 | 1 **AND** 30 | 251 |
| 67 | 1 **AND** 31 | 20 |
| 68 | 1 **AND** 32 | 3 |
| 69 | 1 **AND** 33 | 33 |
| 70 | 1 **AND** 34 | 4 |
| 71 | 1 **AND** 35 | 396 |
| 72 | 1 **AND** 36 | 0 |
| 73 | 1 **AND** 37 | 0 |

# Supplementary Table2

Supplementary appendix2: Characteristics of the studies included

| **First author** | **Year** | **Country** | **Study**  **type** | **Inflammation type** | **Sample (T/C)** | **Mean±SD age,years(T/C)** | **Source** | **Experimental method** | **Variables adjusted** | **NOS score** |
| --- | --- | --- | --- | --- | --- | --- | --- | --- | --- | --- |
| Jie Chen,et al. | 2019 | China | Cross section | AP | 81(44/37) | 56.1 ± 2.3/48.5 ± 2.2 | serum | ELISA | Age,Sex,BMI,Etiology,Organ failure in hospital days,Complications | 7 |
| Pérola Michelle Vasconcelos Caribé,et al.(1) | 2019 | Canada | Cross section | PD | 78(40/38) | 58±8/58±8 | serum | ELISA | Age,Sex,BMI | 7 |
| Pérola Michelle Vasconcelos Caribé,et al.(2a) | 2020 | Canada | Case control | PD | 40(20/20) | 54.2 ± 4.8/56.5 ± 6.7 | serum | ELISA | Age,Sex,BMI | 7 |
| Pérola Michelle Vasconcelos Caribé,et al.(2b) | 2020 | Canada | Case control | PD | 38(18/20) | 62.0 ± 11.1/56.5 ± 6.7 | serum | ELISA | Age,Sex,BMI | 7 |
| Xin Cheng,et al. | 2021 | China | Cross section | Sepsis | 360(180/180) | 53.7±10.9/NR | serum | ELISA | Age,Sex,BMI,Smoking,COPD,Cardiomyopathy,Chronic kidney failure,Cirrhosis,Primary infection site,Primary organism,Biochemical indexes,Disease severity score | 7 |
| X Li,et al. | 2021 | China | Case control | RA | 229(141/88) | 52.7±15.8/52.6±15.2 | serum | ELISA | Age,Sex | 6 |
| Hassan Ghobadi,et al. | 2021 | Iran | Case control | COPD | 60(30/30) | 58.83±9.47/56.27±8.12 | serum | ELISA | Age,Sex,BMI,Pulmonary function test | 6 |
| Chan Yang,et al.(a) | 2022 | China | Cross section | OA | 90(30/60) | NR/42.78 ± 15.20 | plasma | ELISA | NR | 7 |
| Chan Yang,et al.(b) | 2022 | China | Cross section | SLE | 149(89/60) | 42.3 ± 15.44/42.78 ± 15.20 | plasma | ELISA | Age,Sex,Lupus headache,Arthritis,Cylindruria,Hematuria,Proteinuria,Pyuria,Rash,Alopecia,Dental ulcer,Pleuritis,Pericarditis,Hypocomplementemia,Fever | 6 |
| Chan Yang,et al.(c) | 2022 | China | Cross section | Gout | 90(30/60) | NR/42.78 ± 15.20 | plasma | ELISA | NR | 6 |
| Chan Yang,et al.(d) | 2022 | China | Cross section | SS | 98(38/60) | NR/42.78 ± 15.20 | plasma | ELISA | NR | 6 |
| Chan Yang,et al.(e) | 2022 | China | Cross section | AS | 80(20/60) | NR/42.78 ± 15.20 | plasma | ELISA | NR | 6 |
| Chan Yang,et al.(f) | 2022 | China | Cross section | MCTD | 90(30/60) | NR/42.78 ± 15.20 | plasma | ELISA | NR | 6 |
| Chan Yang,et al.(g) | 2022 | China | Cross section | SSc | 72(12/60) | NR/42.78 ± 15.20 | plasma | ELISA | NR | 6 |
| Mirko Manetti,et al. | 2022 | Italy | Cross section | SSc | 151(80/71) | 58.3±13.6/59.7±12.8 | serum | ELISA | Age,Sex,Disease subset,Autoantibody positivity,Digital ulcers,Capillaroscopy pattern | 8 |
| A Saedan Kutin,et al.(a) | 2022 | Iraq | Cross section | ASTH | 60(30/30) | 48.27±1.68/44.567±1.88 | serum | ELISA | Age,Sex,Family history with diseases,Hypertension,Diabetes,Smoking,O2% | 6 |
| A Saedan Kutin,et al.(b) | 2022 | Iraq | Cross section | AR | 70(40/30) | 42.43±1.76/44.567±1.88 | serum | ELISA | Age,Sex,Family history with diseases,Hypertension,Diabetes,Smoking,O2% | 6 |
| Yue Jiang,et al. | 2022 | China | Cross section | AP | 86(43/43) | 55.23±2.25/54.89±2.55 | serum | ELISA | Age,Sex | 6 |
| Liming Tan,et al. | 2023 | China | Cross section | RA | 270(212/58) | 52.87 ± 15.48/50.51 ± 15.35 | serum | ELISA | Age,Sex | 7 |
| Randa Erfan,et al. | 2023 | Egypt | Cross section | Psoriasis | 88(44/44) | 39.3±12.6/39.4±11.2 | plasma | ELISA | Age,Sex,BMI | 6 |
| Chunjuan Chen,et al. | 2023 | China | Case control | AMI | 88(68/20) | 62.94 ± 11.42/60.55 ± 9.89 | plasma | ELISA | Age,Sex,Diabetes mellitus,Hypertension,Current smoker | 8 |
